# Data for increased Rho kinase activity in type 2 diabetic patients

**DOI:** 10.1016/j.dib.2016.11.032

**Published:** 2016-11-16

**Authors:** Lei Liu, Lun Tan, Jinsheng Lai, Sheng Li, Dao Wen Wang

**Affiliations:** Department of Internal Medicine, Tongji Hospital, Tongji Medical College, Huazhong University of Science and Technology, Wuhan, China

## Abstract

The data presented in this article are related to the research article entitled “Enhanced Rho-Kinase Activity: Pathophysiological Relevance in Type 2 Diabetes” [1]. Rho-Kinase has attracted a great deal of interest as a novel therapeutic target in cardiovascular diseases. These data describe the observed relationship of Rho-Kinase activity with type 2 diabetic patients. Rho-Kinase activity is determined by immunoblotting of peripheral blood leukocytes with the Phospho-Thr853 in the myosin-binding subunit of myosin light-chain phosphatase. The level of IL-6 is measured using *Enzyme-linked immunosorbent assay*.

**Specifications Table**

**Table:** 

Subject area	*Biology*
More specific subject area	*Endocrinology*
Type of data	*Figure*
How data was acquired	*Western blot, Enzyme-linked immunosorbent assay*
Data format	*Analyzed*
Experimental factors	*IL-6, Rho-Kinase activity*
Experimental features	Rho-Kinase activity is determined by western blot. The level of IL-6 is measured using *Enzyme-linked immunosorbent assay*.
Data source location	*Hubei, China*
Data accessibility	*Data are within this article*

**Value of the data**

• The stratification of participants according to gender adds further insight to these data.
• These data can be used for further studies on the relationship between IL-6 and Rho-Kinase activity.
• Our data could be useful to characterizing the link between Rho-Kinase and diabetes.

## 1 Data

This article presents figures describing the variation in Rho-Kinase according to gender and the relationship of IL-6 and Rho-Kinase activity, in support of the article [1].

**Fig. 1:**
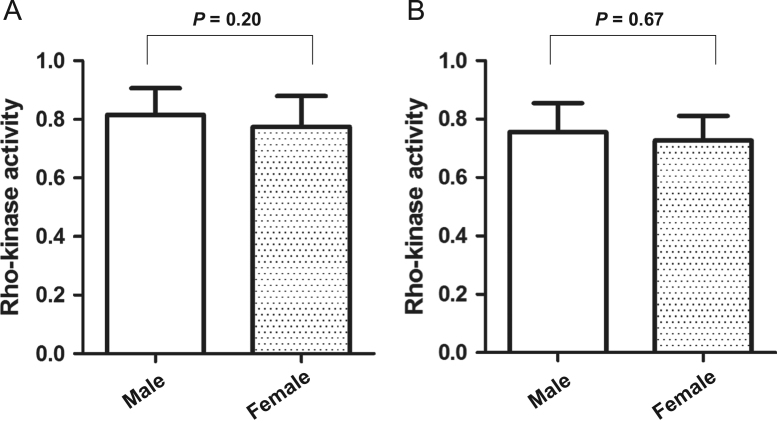
The relationship between Rho-kinase activity and gender. A: Among female type 2 diabetic patients, leukocyte ROCK activity were 0.77±0.10, which is similar to male type 2 diabetic patients (0.81±0.09; P=0.20. B: In control group, ROCK activity was not significantly different between female and male groups (0.72±0.07 vs. 0.73±0.09; P=0.67).

The relationship between Rho-kinase activity and gender is presented in Fig. 1.

**Fig. 2:**
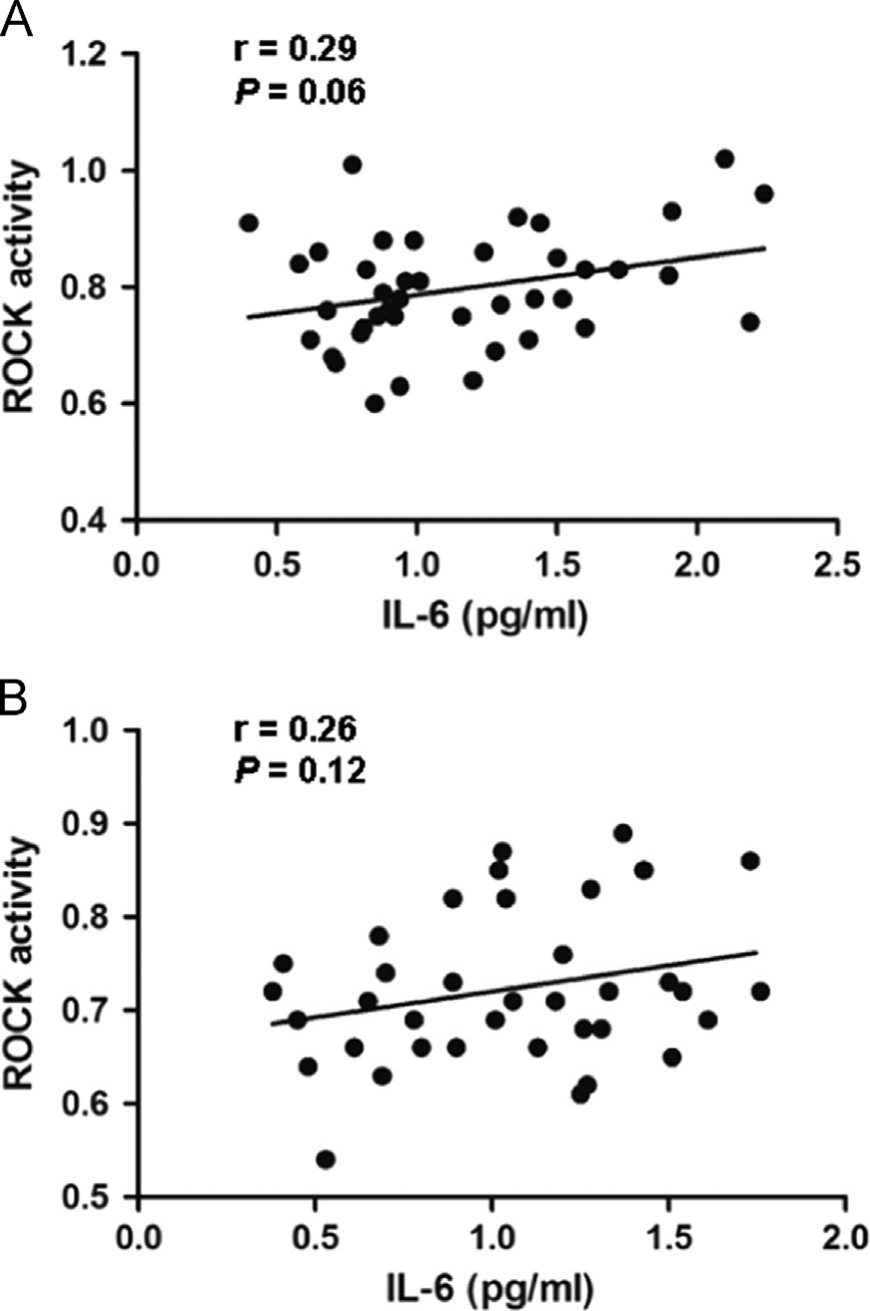
Correlations of ROCK activity with serum IL-6 concentration in type 2 diabetic patients and control subjects. A: In type 2 diabetic patients, a marginal correlation between the level of IL-6 and ROCK activity has been found (r=0.29, P=0.06); B: In control group, no significant correlation between the level of IL-6 and ROCK activity has been found (r=0.26, P=0.12).

Distribution of scatter plot diagrams (Fig. 2) show the association between Rho-kinase activity and IL-6 concentrations.

Seventy-eight volunteers, including 41 type 2 diabetic patients and 37 control subjects, were participated in this study.

## 2 Experimental design, materials and methods

### 2.1 Participants

In the current study, 41 patients with type 2 diabetes and 37 controls were recruited from individuals who admitted the outpatient clinic of Tongji Hospital in Wuhan (Hubei, People׳s Republic of China). Type 2 diabetic patients were confirmed by OGTT according to the American Diabetes Association criteria [2], or by a report of the use of medication for type 2 diabetes, or based on a review of medical records. Patients with type 1 diabetes or gestational diabetes mellitus were excluded on clinical grounds as described previously [3]. The control subjects were determined to be free of type 2 diabetes by their medical history and OGTT test. This study was approved by the institutional review board of Tongji Hospital. Experiments were conducted according to the principles expressed in the Declaration of Helsinki. Written informed consent was obtained from all the participants.

### 2.2 Measurement of ROCK activity in circulating leukocytes

Rho-kinase activity was assessed by measuring the levels of phosphorylated to total myosin-binding subunit (MBS) of myosin light-chain phosphatase, a direct downstream target of Rho-kinase, and by analysis of total Rho-kinase isoforms in circulating leukocytes from venous blood, as previously reported [4], [5], [6], [7], [8], [9]. Fasting venous blood was collected at room temperature in EDTA tubes with fasudil.

To avoid degradation of target proteins by cellular enzymes, proteins must be prepared rapidly in the presence of many protease inhibitors. Furthermore, the culture medium is removed rapidly and completely, and the cells are washed twice with ice-cold phosphate-buffered saline (PBS) before the addition of protease inhibitors and cell fixatives. Meanwhile, scrape the cell contents and transfer them to a microcentrifuge tube on ice. After vortexing and centrifuging the samples at 4° for 5 min over 12,000 rpm, the supernatant is removed and the pellets are collected for Rho-kinase assay. Of note, proteins are stored at −80° until use.

To avoid the interference by different exposure durations and variable membrane conditions, we use lipopolysaccharide-pretreated NIH/3T3 cell lysates as a positive control and also to standardize results between different experiments as suggested [4].

To isolate human leukocytes, all protocols were performed as previously reports [4]. Peripheral blood leukocytes from fasting venous blood samples were isolated by density gradient centrifugation. The leukocyte pellet was resuspended in medium 199 and counted by a hematocytometer. Cells were fixed in 10% trichloroacetic acid and 10 mmol/l dichlorodiphenyltrichloroethane. After centrifugation, cell pellets were dissolved in lysis buffer. Protein content of supernatants was determined by Bradford assay. Equal amounts of leukocyte protein extracts were subjected to 6% sodium dodecyl sulfate-polyacrylamide gel electrophoresis and transferred to PVDF membranes. Membranes were incubated with rabbit anti-phospho-specific Thr853-MBS polyclonal antibody. Bands were visualized using the ECL system. Each blot was quantified by scanning densitometry with the Quantity One software (Bio-Rad, Hercules, CA).ROCK activity is expressed as the ratio of pMBS in each sample per pMBS in each positive control divided by MBS in each sample per MBS in each positive control.

### 2.3 Measurement the serum level of IL-6

The serum level of IL-6 was measured using enzyme immunoassay – the commercially available IL-6 immunoassay system kit according to the manufacturer׳s instructions. To determine the concentrations of circulating IL-6, a standard curve was created. Samples were assayed in duplicate, and IL-6 concentrations were derived from a standard curve composed of serial dilutions of purified recombinant human IL-6. The assay sensitivity was 0.1 pg/ml. The intra-assay coefficient of variation (CV) and inter-assay CV were 3–6% and 4–7%, respectively.

### 2.4 Statistical analysis

The data are expressed as percentages for categorical variables and as mean (standard deviation, SD) or median (interquartile range, IQR) for the continuous variables depending on their normal distribution. Shapiro–Wilk tests were used for normal distribution test. Correlations among continuous variables were assessed by Spearman׳s rank correlation coefficient. Proportions were compared using the chi-squared test, and Student׳s *t*-test or the Mann–Whitney test was used to compare continuous variables between groups as appropriate. All statistical analyses were performed with SPSS for Windows, version 15.0 (SPSS Inc., Chicago, IL, USA). Statistical significance was defined as *P*<0.05.
